# 

**Published:** 1905-11

**Authors:** 


SUBSCRIPTION $1.00.

PUBLISHED MONTHLY.

### ATLANTA JOURNAL-RECORD OF MEDICINE.

NOV. 11 1905

Successor to Atlanta Medical and Surgical Journal, Established 1855,
and Southern Medical Record, Established 1870.

VOL. VII. ATLANTA, GA., NOVEMBER, 1905. No. 8.

